# Imine Palladacycles: Synthesis, Structural Analysis and Application in Suzuki–Miyaura Cross Coupling in Semi-Aqueous Media

**DOI:** 10.3390/molecules27103146

**Published:** 2022-05-14

**Authors:** Brais Bermúdez-Puente, Luis A. Adrio, Fátima Lucio-Martínez, Francisco Reigosa, Juan M. Ortigueira, José M. Vila

**Affiliations:** Department of Inorganic Chemistry, University of Santiago de Compostela, E-15702 Santiago de Compostela, Spain; qibermudez@gmail.com (B.B.-P.); luis.adrio@gmail.es (L.A.A.); fatimaluciomartinez@gmail.com (F.L.-M.); francisco.reigosa@usc.es (F.R.); juanm.ortigueira@usc.es (J.M.O.)

**Keywords:** palladacycles, imines, catalysis, Suzuki, cross coupling

## Abstract

Treatment of the imines **a–c** with palladium(II) acetate in acetic acid yielded the μ-acetate dinuclear complexes **1a–c**, which readily reacted with sodium chloride or bromide to provide μ-halide analogues. The reaction of the latter with nitrogen, phosphorus and oxygen donor nucleophiles yielded new imine palladacycles following the cleavage of the Pd_2_X_2_ unit. The complexes were fully characterized by microanalysis, ^1^H, ^13^C and ^31^P NMR spectroscopies, as appropriate. The compounds were applied as catalysts in the Suzuki–Miyaura coupling reaction in aqueous and semi-aqueous media.

## 1. Introduction

Since the first cyclometallated compound was reported by Cope and Siekman [1], the chemistry of metallacycles dealing in the activation of aromatic C–H bonds by transition metals has attracted much attention. Their development has been extended to a rather large variety of metals, especially palladium [2], and organic ligands, among which Schiff bases, [3] thiosemicarbazones [4] and pincer ligands [5] stand out. The high level of interest in these complexes mainly stems from the high number of applications they provide, such as in metallomesogens [6], as antineoplastic substances [7,8] and in synthetic chemistry, where they have been used to functionalize aromatic carbons through the insertion reactions of molecules such as CO [9], alkenes [10] or alkynes [11]. In the field of catalysis, after the breakthrough with phosphine palladacycles by Hermann et al. [12,13,14], a myriad of palladacycles have been used as pre-catalysts in cross-coupling reactions, such as in the work of Suzuki–Miyaura [15,16,17,18,19], Mizoroki–Heck [20], Negishi [21] and Sonogashira [22]. Although there are commercially available reagents such as [Pd(OAc)_2_] and [Pd(PPh_3_)_4_] that are more than acceptable catalysts, taking into account that many palladium-mediated coupling reactions involve palladacycle intermediates, palladacycles have emerged as a paramount group of catalysts owing, in part, to their stability in air and moisture.

Schiff bases, also known as imines, first prepared by H. Schiff [23] are an extensively used group of ligands, mostly due to the variety of available amines and aldehydes as well as to their ease of preparation, as they are more often than not synthesized by a condensation reaction between organic carbonyl substrate and primary amine; ketones may form imines as well, but the reaction is not so straightforward. They are able to stabilize numerous metals in different oxidation states, controlling the performance of the metals in catalytic processes. They are very suitable for the preparation of imine palladacycles, where they show a rather versatile behavior in terms of the type of metallated carbon atom, whether it be C(sp^2^) or C(sp^3^) ([24]), the choice of the metallation position influenced by the ring substituents [25] or unusual reactivity in the case of bidentate imine ligands [26], providing an extensive group of organometallics. Imine palladacycles are pre-catalysts that yield the appropriate palladium themselves, or, alternatively, appear as intermediates and are one of the choice complexes for Suzuki–Miyaura cross-coupling. One of the most efficient palladating agents for imines is [Pd(OAc)_2_], which renders the μ-acetate dinuclear complex and the μ-chloro analogue after a metathesis reaction. The bridging ligands may be totally or in part substituted to give mono- and dinuclear palladacycles [27,28], which are active as catalysts for Suzuki cross-coupling. For non-cyclometallated compounds, the catalytic activity of Schiff base transition metal complexes and the applications of polymer-supported Schiff base complexes has been reviewed in detail [29,30,31].

The high catalytic activity of dimeric Pd(II) species similar to the μ-acetate complexes described herein for the Heck reaction have demonstrated the efficiency of phosphine-free systems and the often slow activation of the precatalyst. The proposed catalytic mechanisms and kinetic studies highlight the role of water in accelerating the initial preparation of the active catalyst species. Herein, we report a comparative study of the catalytic activity of imine palladacycles derived from the reaction of μ-chloro complexes with a variety of nitrogen, phosphorus and oxygen donor nucleophiles. Nevertheless, the activity of the dinuclear μ-chloro complexes themselves using aqueous mixtures of solvents yields higher results than that of the parent μ-acetato species [32,33]. Also, structures for two and μ-chloro complexes are discussed.

## 2. Results and Discussion

**Scheme 1:**
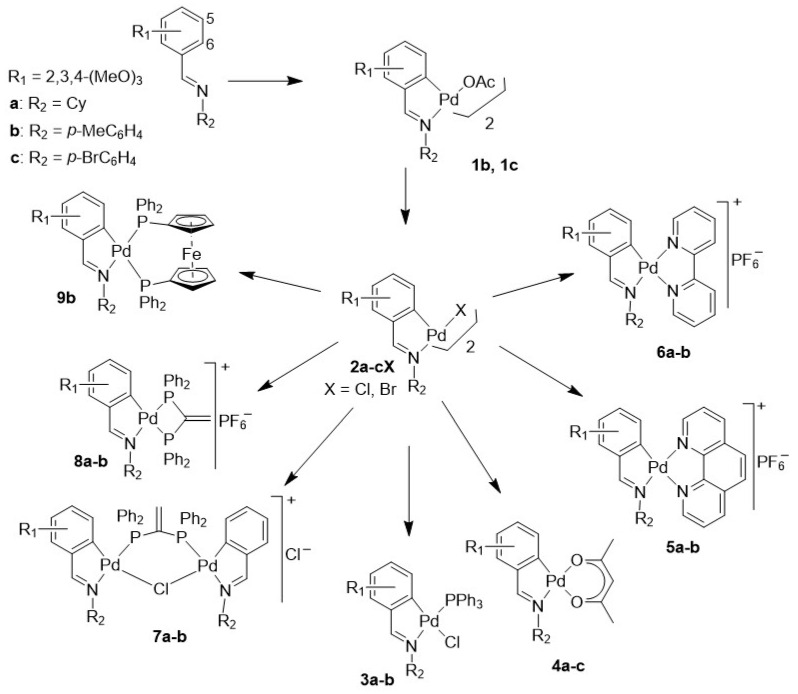
Reactivity sequence for the synthesized compounds.

For the convenience of the reader, the compounds and reactions are shown in Scheme 1. The compounds described in this paper were characterized by elemental analysis (C, H, N), by IR (data in Section 4) and by ^1^H, ^13^C and ^31^P-{^1^H}NMR spectroscopy. (See Experimental Section). The ligands **a**, **b** and **c** were synthesized by mixing the corresponding aldehyde and amine in water following the method by Tanaka and Shiraishi [34]. The treatment of the ligands with palladium(II) acetate in acetic acid at 65 °C for 8 h yielded the μ-acetate dinuclear complexes **1a**-**c**. The coordination of the nitrogen atom to palladium was supported by the shift of the υ(C=N) stretching vibration to a lower frequency in the IR spectra [35] compared to the free ligand spectra and an upfield shift of the HC=N resonance in the ^1^H NMR spectra. [36] The υ_as_(COO) and υ_s_(COO) values were consistent with bridging acetato groups; [37] a singlet resonance in the ^1^H NMR spectra was assigned to the two equivalent acetate groups, set in a *trans* arrangement. The absence of the H6 resonance and strong downfield shift of the C(6) resonance in the ^1^H and ^13^C NMR spectra, respectively, was consistent with the metalation of the phenyl ring at this ring site.

The treatment of complexes **1a**–**c** with aqueous sodium chloride or bromide after a metathesis reaction readily provided the μ-halide complexes **2a**–**cX** (X = Cl, Br), which were fully characterized (see Experimental Section). The spectroscopic data were in agreement with the absence of the μ-acetate ligands.

### 2.1. Crystal Structure of ***1c***

**Figure 1:**
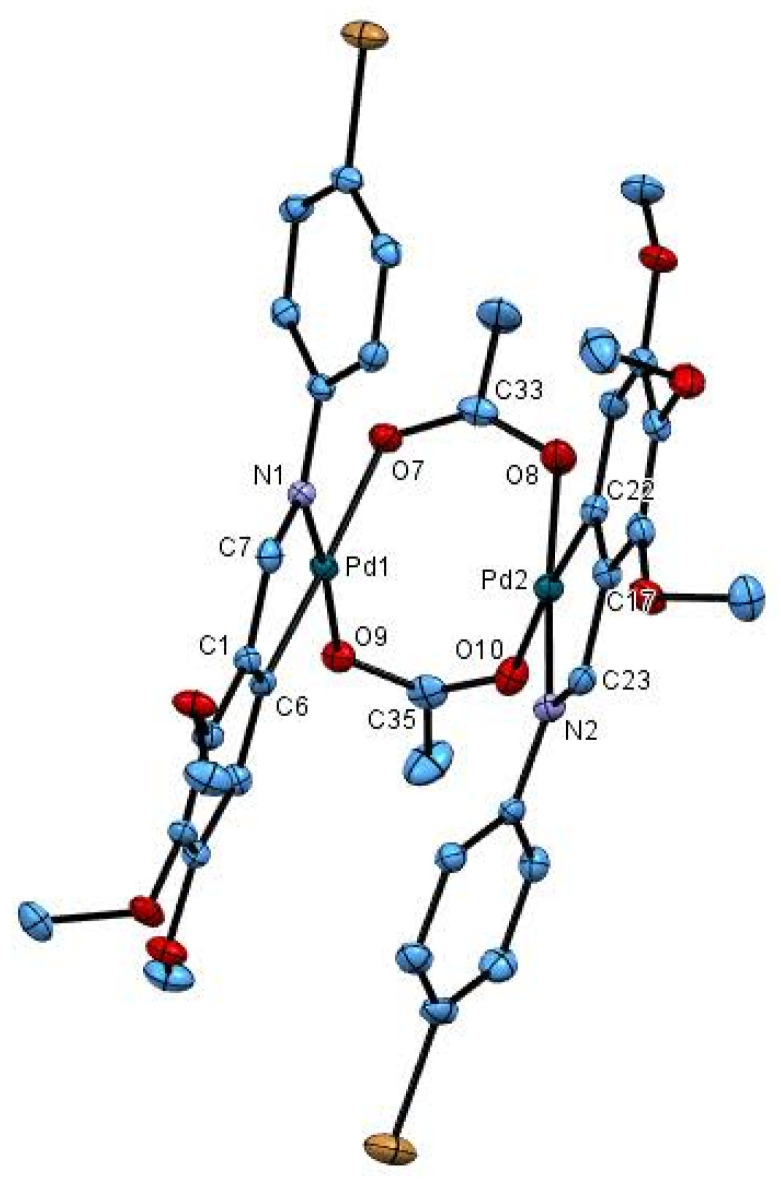
Thermal ellipsoid plot of **1c** shown at the 30% probability level. Hydrogen atoms and angles and minor disorder components have been omitted for clarity. Selected bond lengths (Å) and angles (°): Pd(1)-O(7) 2.140(2); Pd(1)-O(9) 2.045(2); Pd(1)-N(1) 2.027(3); Pd(1)-C(6) 1.962(3); Pd(2)-O(8) 2.041(2); Pd(2)-O(10) 2.141(2); Pd(2)-N(2) 2.045(3); Pd(2)-C(22) 1.962(3); O(7)-Pd(1)-O(9) 89.04(8); N(1)-Pd(1)-O(7) 98.09(9); C(6)-Pd(1)-O(9) 91.70(11); C(6)-Pd(1)-N(1) 81.16(12); O(10)-Pd(2)-O(8) 89.43(9); O(10)-Pd(2)-N(2) 98.08(9); C(22)-Pd(2)-N(2) 81.56(12); C(22) Pd(2) O(8) 90.78(11).

Suitable crystals of **1c** were grown by recrystallization from a dichloromethane-hexane solution. The structure of **1c** is shown in Figure 1, and the Experimental Section and SI contain the crystallographic data. The crystal consists of discrete dinuclear moieties separated by normal van der Waals distances, with a C2 symmetry via a twofold axis at 90° to the Pd–Pd vector. The palladium atoms are coordinated to an *ortho* carbon atom from the phenyl ring, a nitrogen atom from the C=N group and two oxygen atoms from the acetate ligands in distorted square-planar geometry. The imine units are in a *trans* arrangement, recently coined as the “butterfly” geometry, [38] with the metallated imines held together by the bridging acetate ligands. The bond distances and angles are within the reported values, with allowance for the palladium–oxygen distance *trans* to carbon [*cf*. Pd(1)-O(7) 2.140 vs. Pd(1)-O(9) 2.045] due to the *trans*-lengthening influence of the σ-bonded carbon and with a somewhat reduced value for the C(6)-Pd(1)-N(1) and C(22)-Pd(2)-N(2) bond angles, 81.17° and 81.56°, respectively, consequent upon chelation. As for the eight-membered ring formed by the palladium atoms and the acetate ligands in an open-book structure, the [O(8)-C(3)-O(7)] and [O(9)-C(35)-O(10) planes are at an angle of 87.59°, with the palladium coordination planes at 29.19°. The palladium–palladium bond of 2.911 Å may be regarded as non-bonding.

### 2.2. Crystal Structure of ***2cCl***

**Figure 2:**
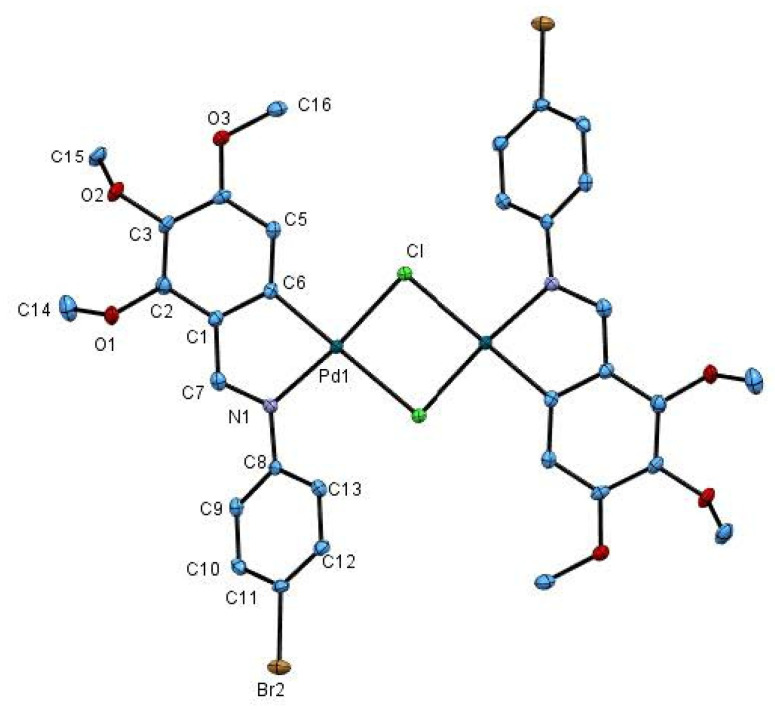
Thermal ellipsoid plot of **2cCl** shown at the 30% probability level. Hydrogen atoms and minor disorder components have been omitted for clarity. Selected bond lengths (Å) and angles (°): Pd(1)-Cl(1) 2.4485(8); Pd(1)-Cl(1) 2.3338(8); Pd(1)-N(1) 2.048(3); Pd(1)-C(6) 1.966(3); N(1)-Pd(1)-Cl(1) 173.79(7); N(1)-Pd(1)-Cl(1) 99.35(7); C(6)-Pd(1)-Cl(1) 93.83(9); C(6)-Pd(1)-Cl(1) 178.38(9); C(6)-Pd(1)-N(1) 81.20(11); Cl(1)-Pd-(1)Cl(1) 85.52.

Single crystals of complex **2cCl** suitable for X-ray analysis were grown by slowly evaporating an n-hexane–chloroform solution at room temperature. The molecular structure is shown in Figure 2, and crystal data are in the Section 4 and Supplemetnary Materials.

The asymmetric unit comprises half of the molecule with a crystallographic inversion center located at the center of the Pd_2_(μ-Cl)_2_ moiety. The coordination sphere around each palladium atom consists of two halogen atoms, a C=N nitrogen atom and the C(6) carbon atom. The coordination environment at the metal center is distorted square planar, with the most noticeable deviation corresponding to the C-Pd-N bite angle of 81.29(13).

The [(C-N)Pd(μ-Cl)_2_Pd(C-N)] fragment adopts a planar configuration, with an angle between the palladium coordination plane and the Pd_2_Cl_2_ ring of 6.49°. This situation is the most common configuration observed in related species [39]. All bond distances are within the expected values, with allowance for the lengthening of the Pd–X bond *trans* to carbon, due to the differing *trans* influence of the carbon and nitrogen atoms, resulting in an asymmetric Pd(μ-X)_2_Pd moiety.

The treatment of the halide-bridged complexes with the nitrogen, phosphorus or oxygen donor ligands 1,1′-bipyridine, 1,10-phenantroline, triphenylphosphine, 1,1-bis(diphenylphosphine)ethene, 1,1′-bis(diphenylphosphine)ferrocene and acetylacetonate in the appropriate molar ratio yielded the corresponding mono- and dinuclear air-stable solids, which were fully characterized (See Scheme 1 and the Experimental Section). For **4a–c** the ^1^H NMR spectra show singlet resonances ca. 5.3, and 2.0 and 1.9 ppm, respectively, assigned to the CH and to the two non-equivalent methyl protons, also respectively. The ^1^H NMR for the phosphine derivatives showed an upfield shift in the 4-MeO group ca. 0.8 ppm promoted by the shielding of the phosphine phenyl rings; this agrees with a N-Pd-P trans geometry in **3a–b** and with the parallel arrangement of the metalated moieties in **7a–b**, as shown in Scheme 1, again with a phosphorus *trans* to nitrogen geometry. The ^31^P NMR spectra showed a singlet resonance for the two equivalent ^31^P nuclei in **7a–b** and two doublets for **8a–b**, **9b**; the lower frequency doublet was assigned to the phosphorus nucleus *trans* to the phenyl carbon, C(6), and the higher frequency doublet to the phosphorus nucleus *trans* to the imine nitrogen, based on the assumption that a ligand of greater *trans* influence shifts the ^31^P resonance in *trans* to lower frequency [40].

### 2.3. Catalytic Activity

To test the catalytic activity of the new imine palladacycles depicted herein, they were probed as potential catalysts in Suzuki–Miyaura coupling (SMC) to render the corresponding biaryl species. In order to attain a clear picture of the catalytic potential of the mentioned compounds, all were tested for a standard coupling reaction in order to stablish the most efficient catalysts. Thus, the treatment of 4-bromoacetophenone with phenylboronic acid in THF:water (2:1) at room temperature (rt) or at 80 °C for a maximum of 24 h in the presence of 2 mol% catalyst and base, K_2_CO_3_, gave the biphenyl-coupled product 4-phenylacetophenone at >80% in the majority of cases (Table 1). The use of a solvent different from the one stated above gave poorer results.

With regard to the results of this study, it can be concluded that the compounds with bpy and phen ligands, entries 22–30, in general show a much lower level of activity than the compounds tested. This may be due to the strong chelating nature of the nitrogen donor, i.e., the complex formed with the palladacycles unit is highly stable, which hinders its interaction with the substrates; this is reflected in a lower catalytic activity. In most cases the reactions at room temperature give a lower conversion than those performed at 80 °C. This is not the case for compounds with bridging chlorine and bromine ligands, entries 3–9, in which high activity is observed both at 80 °C and at room temperature, which led us to choose this type of compound as the best performers. Compounds with triphenylphosphine, entries 10 and 12, also appeared to show good results at both temperatures. As for compounds with bridging or chelating diphosphine, high conversions could be obtained at room temperature, but the reaction time had to be extended to 24 h (entries 32 and 34). Heating to 80 °C brings down the reaction time to only 5 h (entries 31, 33, 35, 36). Some compounds with acetylacetonate ligands (entries 17–19) follow a similar pattern, but the others (entries 20, 21) gave low yields ca. 50%. We propose that the halide-bridge species, which show yields of 80–100% (entries 3–9), should be singled out as the most efficient compounds as catalysts; given their straightforward synthesis and stability, they are the group of choice to carry out a more detailed study of catalytic activity, and among them we selected **2bCl**, for which case the conditions for its catalytic activity could be narrowed to provide a yield of 100% at room temperature in two of the conditions tested; the results are shown in Table 2.

For the experiments carried out at 80 °C (entries 1–4), the better results were obtained using THF:H_2_O or EtOH:H_2_O mixtures; even water itself gave a reasonably acceptable output (entry 3). At room temperature (entries 5–8), the solvent of choice appeared to be the EtOH:H_2_O mixture; no significant differences in conversion were observed when bases such as K_2_CO_3_ or K_3_PO_4_ were used. In view of the results, EtOH:H_2_O as medium and K_2_CO_3_ as base were selected for further experiments, as they were the most environmentally conscious and less expensive options, respectively.

Hence, under the conditions in entry **7** (Table 2), the cross couplings for the different aryl and benzyl halides with phenylboronic acid were carried out, catalyzed by compound **2bCl,** as shown in Table 3. The results were satisfactory for the majority of cases. The reaction comes to completion with the different aryl bromides, having both activating (entries 6, 7 and 8) and deactivating (entry 5) groups, even at room temperature and in short reaction times. Where a chlorine atom is also present (entry 14), the coupling is selective on the bromine atom and the carbon–chlorine bond remains unchanged under the conditions indicated. The reaction was also efficient with bromide not directly bonded to the aromatic carbon (entry 9), giving rise to the coupling reaction of benzyl bromides. In the case of entries 10–13, the selectivity between aromatic bromide and benzyl bromide was studied. Treatment with an equivalent of the boronic acid to obtain the compound coupled to the aromatic carbon gave a mixture in which the desired compound was the major one, but the product coupled through the benzyl bromide was also produced in a lower proportion (entries 10 and 11). Reaction with two equivalents of the boronic acid, in the hope of preparing a doubly coupled compound, gave a mixture of products as well, i.e., **10** and **12** (entries 12 and 13). As for the coupling of aryl chlorides, in the case of starting compounds with activating groups, the reaction takes place almost quantitatively at a high temperature (entry 1), while at room temperature the product is obtained with moderate yields (entries 2–3). In the case of using deactivated chlorides such as 4-chloroanisole, the reaction hardly takes place at all (entry 4).

In order to try to optimize the reaction with 4-chloroanisole, different additional ligands were added to the reaction, such as dppf and triphenylphosphine. With the first ligand, no positive result was obtained and the inhibition of the performance of the catalyst was observed. Meanwhile, with the addition of triphenylphosphine 5% molar, the conversion of the coupling reaction increased slightly from 13% (entry 4) to 30%, but a by-product was also formed which could not be identified.

The coupling reaction with chloride derivatives and **2bCl** as catalyst was compared with palladium acetate, under the same conditions. The results obtained are shown in Table 4.

It can be seen that the **2bCl** catalyst provides better conversions than palladium acetate per palladium atom used. Furthermore, ligand **b** appears to play an important role in the catalytic activity of the catalyst; if added to palladium acetate, the yield of the reaction improves, which could be because it slows the formation of Pd(0) aggregates.

## 3. Conclusions

We have shown that imine palladacycles may be prepared in good yield from palladium(II) acetate and the appropriate Schiff base ligand to provide μ-acetate complexes, which undergo metathesis reactions to yield μ-halide analogues. The latter readily undergo bridge splitting reactions to provide the corresponding mononuclear species, with the exception of μ-diphosphine ligands, in which case dinuclear compounds may also be formed. The resulting palladacycles were tested for Suzuki–Miyaura coupling by varying the reaction conditions such as the base, time and temperature, to conclude that the μ-halide complexes appeared to show the best yields. We suggest this could be due to the ease with which the Pd_2_X_2_ moiety may be cleaved and the halide ligand either substituted or removed from the palladium coordination environment, facilitating the potential of palladacycle as a pre-catalyst. Among these, the compound labelled **2bCl** was then applied to the SMC of aryl halides and phenylboronic acid in different aqueous mixtures, providing good conversions; **2bCl** also showed a catalytic activity greater than the standard palladium(II) acetate under the same conditions, suggesting a promising future for μ-halide imine palladacycles as catalysts for the SMC. Also, in the light of these results, further studies shall be conducted to determine if it is possible that other palladacycles included in Table 1 can be applied to the substrates in Table 3, especially with these being more sterically demanding as well as bearing different +M/−M/+I/−I substituents, especially if the precatalyst activation is the rate limiting step.

## 4. Experimental Section

*X-ray structure determination*. Crystallographic data of the structures described in this work were collected on a Bruker Kappa APEX II diffractometer (Mo Kα radiation, λ = 0.71073 Å) equipped with a graphite monochromator by the method of the ω and φ scans at 293 K, integrated and corrected for absorption and solved and refined using routine techniques. All non-hydrogen atoms were refined anisotropically; hydrogen atoms were included in calculated positions and refined in riding mode.

*General Procedures*. Solvents were used without previous purification. Chemicals were reagent grade. The diphosphine Ph_2_PC(=CH_2_)PPh_2_ (vdpp) was purchased from Sigma-Aldrich. Elemental analyses were carried out on a THERMO FINNIGAN, model FLASH 1112. IR spectra were recorded with a JASCO FT/IR–4600 spectrometer equipped with an ATR, model ATR–PRO ONE. ^1^H NMR spectra in solution were recorded in CDCl_3_ or Acetone-d_6_ at room temperature on a Varian Inova 400 spectrometer operating at 300.14 MHz. ^31^P-{1H} NMR spectra were recorded at 202.46 MHz on a Bruker AMX 500 spectrometer. All chemical Shifts are reported downfield from standards, TMS using the solvent signal (CDCl_3_, δ^1^H = 7.26 ppm and Acetone-d_6_ δ^1^H = 2.09) as reference and for ^31^P relative to external H_3_PO_4_ (85%). All the NMR experiments were performed using 5 mm o.d. tubes.

### 4.1. Preparation of the Ligands and Complexes

**Preparation of a–c.** 2,3,4-trimetoxybenzaldehyde (0.5 g, 2.55 mmol1 eq.) and the corresponding amine (2.55 mmol, 1 eq.) were added in water and stirred for 4 h at room temperature. The precipitate was washed with water and dried under vacuum, to give a white solid. **a**: Yield 85%. IR (KBr) υC=N 1633 cm^−1^. ^1^H NMR (CDCl_3_, 300 MHz) δ 8.55 (1H, s, *H*C=N), 7.67 (1H, d, ^3^*J*(H6H5) = 8.94 Hz, H6), 6.7 (1H, d, *J*(H5H6) = 8.92 Hz, H5), 3.92 (3H, s, OMe), 3.89 (3H, s, 3H, OMe), 3.87 (3H, s, OMe), 3.18 (1H, m, N-C*H*Cy), 1.84–1.21 (10H, m, Cy). Anal. found: C: 69.3; H: 8.4; N: 5.08%, C_16_H_23_NO_3_ requires: C: 69.3; H: 8.4; N: 5.05%. **b**: Yield 82%. IR (KBr): υC=N: 1618 cm^−1^. ^1^H NMR (CDCl_3_, 300 MHz) δ 6.77 (1H, d, ^3^*J*(H5H6) = 8.8 Hz, H5), 3.96 (3H, s, OMe), 3.92 (3H, s, OMe), 3.90 (3H, s, OMe), 2.37 (3H, s, Me). Anal. found: C: 70.3; H: 6.8; N: 4.9%, C_17_H_19_NO_3_ requires: C: 71.5; H: 6.7; N: 4.9%. **c**: Yield 93%. IR (KBr): υC=N 1614 cm^−1^. ^1^H NMR (300 MHz, CDCl_3_) δ 8.69 (1H, s, *H*C=N), 7.87 (1H, d, ^3^*J*(H6H5)=8.9 Hz, H6), 7.49 (2H, d, *N* = 8.6 Hz, Hb), 7.08 (2H, d, *N* = 8.6 Hz, Ha), 6.77 (1H, d, *J*(H5H6) = 8.9 Hz, H5), 3.97 (3H, s, OMe), 3.93 (3H, s, OMe), 3.89 (3H, s, OMe). ^13^C (CDCl_3_, 300 MHz) δ 156.69 (C, C-4), 156.27 (CH, HC=N), 154.62 (C, C-2),151.60 (C, C-3), 141.66 (C, C-4′), 131.97 (CH, C-3′, C-5′), 124,1 (C, C-1′), 122.60 (CH, C-2′,C-6′), 118.73 (CH, C-6), 116.55 (C, C-1), 107.71 (CH, C-5), 61.91 (CH, OCH_3_), 60.83 (CH, OCH_3_), 56.00 (CH, OCH_3_). Anal. found: C: 54.7; H: 4.5; N: 3.9%, C_16_H_16_BrNO_3_ requires: C: 54.9; H: 4.6; N: 4.0%.

**Preparation of 1a–c.** Ligand **a**–**c** (0.72 mmol, 1.1 eq.) and palladium acetate (0.65 mmol, 1 eq.) were introduced in a Schlenk flask and vacuum-nitrogen cycles were performed, upon which 25 cm^3^ of deoxygenated acetic acid was injected with a syringe. The resulting solution was stirred at 65 °C for 8 h. Palladium (0) was removed from the mixture by centrifugation and the solution was extracted with dichloromethane and water. The organic layers were collected and dried with sodium sulphate and the solvent was removed under vacuum, resulting in a yellow solid. **1b**: Yield 73%. IR (KBr) υC=N 1593 cm^−1^. ^1^H NMR (CDCl_3_, 300 MHz) δ 7.78 (2H, s, *H*C=N), 6.96 (4H, d, *N* = 8.0 Hz, C_6_H_4_), 6.70 (4H, d, *N* = 8.0 Hz, C_6_H_4_), 5.73 (2H, s, H5), 3.93 (6H, s, OMe), 3.87 (6H, s, OMe), 3.49 (6H, s, OMe), 2.31 (s, 6H, Me), 1.86 (6H, s, Me[Ac]). IR (KBr): υC=N: 1593 cm^−1^, υ_as_COO: 1570 cm^−1^, υ_s_COO: 1411 cm^−1^ Anal. found: C: 52.6; H: 5.1; N: 2.9%. C_38_H_42_N_2_O_10_Pd_2_ requires: C: 50.7; H: 4.7; N: 3.1%. **1c**: Yield 92%. IR (KBr) υC=N 1579 cm^−1^, υ_as_COO: 1570 cm^−1^, υ_s_COO: 1412 cm^−1^. ^1^H NMR (CDCl_3_, 300 MHz) δ 7.83 (2H, s, *H*C=N), 7.28 (4H, d, *N* = 8.8 Hz, Hb), 6.67 (4H, d, *N* = 8.8 Hz, Ha), 5.72 (2H, s, H5), 3.97 (6H, s, OMe), 3.88 (6H, s, OMe), 3.57 (6H, s, OMe), 1.91 (6H, s, OAc). ^13^C NMR (CDCl_3_, 300 MHz) δ 180.34 (CO, Me*C*OO), 169.15 (CH, HC=N), 156.08 (C, C-4), 152.31 (C, C-2), 147.13 (C, C-3), 137.81 (C, C-4′), 131.00 (C, C-3′, C-5′), 130.77 (CH, C-6), 124.86 (CH, C-2′, C-6′), 122.94 (C, C-1′), 120.45 (C, C-1), 110.46 (CH, C-5), 62.20 (CH, OCH_3_), 61.20(CH, OCH_3_), 55.71 (CH, OCH_3_), 24.42 (CH, *Me*COO). Found: C: 44.1; H: 3.6; N: 2.7%. C_36_H_36_Br_2_N_2_O_10_Pd_2_ requires: C: 42.0; H: 3.5; N: 2.7%.

**Preparation of 2bCl-Br**. The halide-bridged compounds were synthesized by adding 15 cm^3^ of a 0.05 M aqueous solution of the corresponding sodium salt to an acetone solution of **1a–c** (0.5 eq.), upon which a precipitate was observed immediately. The solid was filtered off and dried under vacuum. **2aCl**: Yield 67%. IR (KBr) υC=N 1597 cm^−1^, υPd-Cl 333.9/278.4 cm^−1^. ^1^H NMR (CDCl_3_, 300 MHz) δ 7.94 (2H, s, *H*C=N), 6.71 (2H, s, H5), 3.93 (6H, s, OMe), 3.87 (6H, s, OMe), 3.76 (6H, s, OMe), 3.55 (2H, m, NCHCy), 2.2–1.22 (20H, m, Cy). Anal. found: C, 45.9; H, 4.8; N, 3.1%. C_32_H_44_Cl_2_N_2_O_6_Pd_2_ requires: C, 45.9; H, 5.2; N, 3.3%. **2aBr**: Yield 69%. IR (KBr) υC=N: 1598 cm^−1^. ^1^H NMR (CDCl_3_, 300 MHz) δ(ppm): 7.99 (2H, s, *H*C=N), 6.81 (2H, s, H5), 3.93 (6H, s, OMe), 3.88 (6H, s, OMe), 3.76 (6H, s, OMe), 3.55 (2H, m, NCHCy), 2.3–1.04 (20H, m, Cy). Anal. found: C, 41.3; H, 4.5; N, 2.9%. C_32_H_44_Br_2_N_2_O_6_Pd_2_ requires: C, 41.5; H, 4.8; N, 3.0%. **2bCl**: Yellow solid, yield 74%. IR (KBr) υC=N 1578 cm^−1^, υPd-Cl 321.0/275.6 cm^−1^. ^1^H NMR (CDCl_3_, 300 MHz) δ(ppm): 8.04 (2H, s, *H*C=N), 7.25 (4H, d, *N* = 8.1 Hz, C_6_H_4_), 7.14 (4H, d, *N* = 8.2 Hz, C_6_H_4_), 6.59 (2H, s, H5), 3.94 (6H, s, OMe), 3.84 (6H, s, OMe), 3.77 (6H, s, OMe), 2.35 (6H, s, Me). Anal. found: C: 47.4; H: 4.4; N: 3.1. C_34_H_36_Cl_2_N_2_O_6_Pd_2_ requires: C: 47.9; H: 4.2; N: 3.3%. **2bBr**: Yellow solid, yield 70%. IR (KBr) υC=N 1577 cm^−1^. ^1^H NMR (CDCl_3_, 300 MHz) δ(ppm): 8.04 (2H, s, *H*C=N), 7.15 (8H, m, C_6_H_4_), 6.49 (2H, s, H5), 3.94 (6H, s, OMe), 3.84 (6H, s, OMe), 3.77 (6H, s, OMe), 2.32 (6H, s, Me). Anal. found: C, 44.5; H, 4.5; N, 2.7%. C_34_H_36_Br_2_N_2_O_6_Pd_2_ requires: C, 43.4; H, 3.8; N, 2.9%. **2cCl**: Yellow solid, yield 88%. IR (KBr) υC=N 1578 cm^−1^, υPd-Cl: 358.2/281.0 cm^−1^. ^1^H NMR (CDCl_3_, 300 MHz) δ(ppm): 7.88 (2H, s, *H*C=N), 7.30 (4H, d, *N* = 7.7 Hz, Hb), 7.05 (4H, d, *N* = 7.7 Hz, Ha), 6.37 (2H, s, H5), 3.79 (6H, s, OMe), 3.69 (6H, s, OMe), 3.60 (6H, s, OMe). Anal. found: C, 40.0; H, 3.1; N, 2.7%. C_32_H_30_Br_2_Cl_2_N_2_O_6_Pd_2_ requires: C, 39.1; H, 3.1; N, 2.8%. **2cBr**: Yellow solid, yield 90%. IR (KBr) υC=N 1577 cm^−1^. ^1^H NMR (CDCl_3_, 300 MHz) δ(ppm): 8.07 (2H, s, *H*C=N), 7.48 (4H, d, *N* = 8.3 Hz, Hb), 7.20 (4H, d, *N* = 8.3 Hz, Ha), 6.75 (2H, s, H5), 3.95 (6H, s, OMe), 3.87 (6H, s, OMe), 3.77 (6H, s, OMe). Anal. found: C, 37.1; H, 2.9; N, 2.5%. C_32_H_30_Br_4_N_2_O_6_Pd_2_ requires: C, 35.9; H, 2.8; N, 2.6%.

**Preparation of 3a-bCl-Br**. In a round bottom flask, **2a–bCl** or **2a–bBr** (0.060 mmol, 1 eq.) was added in 10 cm^3^ acetone. Triphenylphosphine was added (0.120 mmol, 2 eq.) and the mixture was stirred for 4 h at room temperature. The solvent of the resulting solution was removed under vacuum and the solid was recrystallized from dichloromethane-hexane. **3aCl**: Orange solid, yield 82%. IR (KBr) υC=N 1569 cm^−1^, υPd-Cl 298 cm^−1^. ^1^H NMR (CDCl_3_, 300 MHz) δ 8.33 (1H, d, ^4^*J*(HiP) = 9.0 Hz, *H*C=N), 7.87–7.19 (15H, m, PPh_3_), 5.71 (1H, d, ^4^*J*(H5P) = 6.3 Hz, H5), 4,38 (1H, bs, N-C*H*Cy), 3.90 (3H, s, -OMe), 3.66 (3H, s, -OMe), 2.77 (3H, s, -OMe), 2.40–1.01 (10H, m, Cy). ^31^P NMR (CDCl_3_, 202 MHz) δ 40.90. Anal. found: C, 60.3; H, 6.0; N: 2.1%. C_34_H_37_ClNO_3_PPd requires: C, 60.0; H, 5.5; N, 2.1%. **3aBr**: Yellow solid, yield 68%. IR (KBr) υC=N 1577 cm^−1^. ^1^H NMR (CDCl_3_, 300 MHz) δ 8.37 (1H, d, ^4^*J*(HiP) = 9.1 Hz, *H*C=N), 7.89–7.32 (15H, m, PPh_3_), 5.71 (1H, d, ^4^*J*(H5P) = 6.9 Hz, H5), 4.64 (1H, bs, N-C*H*Cy), 3.93 (3H, s, OMe), 3.69 (3H, s, OMe), 2.80 (3H, s, OMe), 2.40–1.01 (10H, m, Cy). ^31^P NMR (CDCl_3_, 202 MHz) δ 40.80s. Anal. found: C, 56.6; H, 5.3; N: 1.8%. C_34_H_37_BrNO_3_PPd requires: C, 56.3; H, 5.1; N, 1.9%. **3bCl**: Yellow solid, yield 72%. IR (KBr) υC=N 1571 cm^−1^, υPd-Cl 288 cm^−1^. ^1^H NMR (CDCl_3_, 300 MHz) δ 8.44 (1H, s, *H*C=N), 7.86–7.30 (15H, m, PPh_3_), 7.24–7.13 (4H, m, C_6_H_4_), 5.82 (1H, s, H5), 3.92 (3H, s, OMe), 3.71 (3H, s, OMe), 2.87 (3H, s, OMe), 2. 32 (s, 3H, Me). ^31^P NMR (CDCl_3_, 202 MHz) δ(ppm): 40.75s. Anal. found: C: 60.3; H: 4.5; N, 1.7%, C_35_H_33_ClNO_3_PPd requires C, 61.0); H, 4.8; N, 2.0%. **3bBr**: Orange solid, yield 77%. IR (KBr) υC=N 1577 cm^−1^. ^1^H NMR (CDCl_3_, 300 MHz) δ 8.43 (1H, d, ^4^*J*(H_i_-P) = 7.3 Hz, *H*C=N), 7.89–6.97 (15H, m, PPh_3_), 7.16 (4H, m, C_6_H_4_), 5.75 (1H, d, ^4^*J*(H5P) = 6.8 Hz, H5), 3.90 (3H, s, OMe), 3.68 (3H, s, OMe), 2.83 (3H, s, OMe), 2.30 (3H, s, Me). ^31^P NMR (CDCl_3_, 202 MHz) δ 41.69s. Anal. found: C, 57.0; H, 4.3; N: 1.6%. C_35_H_33_BrNO_3_PPd requires: C, 57.3; H, 4.5; N, 1.9%.

**Preparation of 4a–c**. Thalium acetylacetonate (0.12 mmol, 2 eq.) was added to a solution of **2a–cCl** in chloroform (0.06 mmol, 1 eq.). The mixture was stirred for 24 h, after which the precipitate was eliminated by centrifugation and the remaining solution was dried. The residue was purified by chromatography with chloroform as an eluent. This yielded a yellow solid. **4a**: Brown solid, yield 81%. IR (KBr) υC=N 1581 cm^−1^, υ(CO) 1563/1398 cm^−1^, υC=C 1520 cm^−1^. ^1^H NMR (CDCl_3_, 300 MHz) δ(ppm): 8.01 (1H, s, *H*C=N), 6.80 (1H, s, H5), 5.32 (1H, s, Hacac), 3.92 (3H, s, OMe), 3.90 (3H, s, Ome), 3.75 (3H, s, Ome), 3.56–3.41 (1H, m, N-C*H*Cy), 2.29–0.97 (10H, m, Cy). Anal. found: C, 52.3; H, 6.1; N, 3.0%. C_21_H_29_NO_5_Pd requires: C, 52.0; H, 6.3; N, 2.9%. **4b**: Yellow solid, yield 62%. IR (KBr) υC=N: 1586 cm^−1^, υ(CO) 1575/1399 cm^−1^, υC=C 1515 cm^−1^. ^1^H NMR (CDCl_3_, 300 MHz) δ 8.22 (1H, s, *H*C=N), 7.30 (2H, d, *N* = 8.4 Hz, C_6_H_4_), 7.17 (2H, d, *N* = 8.1 Hz, C_6_H_4_), 6.91 (1H, d, H5), 5.34 (1H, s, Hacac), 3.98 (3H, s, Ome), 3.96 (3H, s, Ome), 3.80 (3H, s, Ome), 2.37 (3H, s, Me), 2.07 (3H, s, Me[acac]), 1.88 (3H, s, Me[acac]). Anal. found: C, 53.9; H, 5.1 N, 2.9%. C_22_H_25_NO_5_Pd requires: C, 53.6; H, 5.5; N, 2.8%. **4c** Orange solid, yield 79%. IR (KBr) υC=N 1582 cm^−1^, υ(CO) 1582/1395 cm^−1^, υC=C 1511 cm^−1^. ^1^H NMR (CDCl_3_, 300 MHz) δ(ppm): 8.23 (s, 1H, *H*C=N), 7.50 (2H, d, *N* = 8.9 Hz, Hb), 7.30 (2H, d, *N* = 8.9 Hz, Ha), 6.90 (1H, s, H5), 5.36 (1H, s, *H*acac), 4.01 (3H, s, OMe), 3.98 (3H, s, OMe), 3.81 (3H, s, OMe), 2.08 (s, 3H, Me[acac]), 1.89 (s, 3H, Me[acac]). Anal. found: C, 48.7; H, 5.0 N, 2.2%. C_21_H_22_BrNO_5_Pd requires: C, 45.5; H, 4.2; N, 2.5%.

**Preparation of 5**–**6a–c**. Compounds **5a–c** and **6a–c** were prepared by the reaction of **2a-cCl** (0.060 mmol, 1 eq.) with the corresponding ligand (0.060 mmol, 1 eq.) in dry methanol under nitrogen. The reagents were stirred together for 30 min and then NH_4_PF_6_ was added, triggering the formation of a yellow solid that was filtered off and washed with cold methanol and dried under vacuum. **5a:** Yellow solid, yield 85%. IR (KBr): υC=N 1569 cm^−1^. ^1^H NMR (CDCl_3_, 300 MHz) δ(ppm): 8.81–8.20 (9H, m, bpy, *H*C=N), 6.29 (1H, s, H5), 4.02 (3H, s, OMe), 3.88 (3H, s, OMe), 3.82 (3H, s, OMe), 3.64(1H, m, N-C*H*Cy), 2.32–1.24 (10H, m, Cy). ^31^P NMR (CDCl_3_, 202 MHz) δ-143.23 (q, ^1^*J_P-F_* = 710.74Hz). Anal. found; C, 45.5; H, 4.4; N, 6.0%, C_26_H_30_F_6_N_3_O_3_PPd requires: C, 45.7; H, 4.4; N, 6.1%. **5b**: Yellow solid, yield 80%. IR (KBr) υC=N 1574 cm^−1^. ^1^H NMR (CDCl_3_, 300 MHz) δ(ppm): 8.7–7.7 (8H, m, bpy), 8.49 (1H, s, *H*C=N), 7.58 (2H, d, *N* = 8.4 Hz, C_6_H_4_), 7.40 (2H, d, *N* = 8.4 Hz, C_6_H_4_), 6.71 (1H, s, H5), 4.06 (3H, s, OMe), 4.01 (3H, s, OMe), 3.83 (3H, s, OMe), 2.44 (3H, s, Me). ^31^P NMR (CDCl_3_, 202 MHz) δ-149.67 (q, ^1^*J_P-F_* = 708.44 Hz). C_27_H_26_F_6_N_3_O_3_PPd requires: C, 46.8; H, 3.8; N, 6.0%. **5c**: Yellow solid 89%. IR (KBr) υC=N 1587 cm^−1^. ^1^H NMR (CDCl_3_, 300 MHz) δ(ppm): 9.01–6.40 (9H, m, bpy, *H*C=N) 7.72 (4H, m, Ha, Hb), 6.81 (1H, s, H5), 4.08–4.07 (6H, s, OMe), 3.85 (3H, s, OMe). ^31^P NMR (CDCl_3_, 202 MHz) δ-145.83 (q, *^1^J_P-F_* = 715.20 Hz). Anal. found; C, 43.7; H, 3.5; N, 4.9%, C_26_H_23_BrF_6_N_3_O_3_PPd requires: C, 41.3; H, 3.1; N, 5.5%. **6a**: Yellow solid, yield 83%. IR (KBr) υC=N 1568 cm^−1^. ^1^H NMR (Acetone-d_6_, 300 MHz) δ 9.34–8.27 (9H, m, phen, *H*C=N), 6.42 (1H, s, H5), 4.03 (3H, s, OMe), 3.92 (3H, s, OMe), 3.82 (3H, s, OMe), 2.87 (1H, m, NC*H*Cy), 2.32–1.25 (10H, m, Cy). ^31^P NMR (acetone-d_6_, 202 MHz) δ-145.81 (q, *^1^J_P-F_* = 769.70 Hz). Anal. found; C, 46.4; H, 4.2; N, 5.8%, C_28_H_30_F_6_N_3_O_3_PPd requires: C, 47.5; H, 4.3; N, 6.0%. **6b**: Yellow solid, yield 85%. IR (KBr) υC=N 1573 cm^−1^. ^1^H NMR (CDCl_3_, 300 MHz) δ(ppm) = δ 9.0–7.0 (8H, m, phen), 8.51 (1H, s, *H*C=N), 7.55 (2H, d, *N* = 8.8 Hz, Hb), 7.40 (2H, d, *N* = 8.8 Hz, Ha), 6.79 (1H, s, H5), 4.07 (3H, s, OMe), 4.06 (3H, s, OMe), 3.84 (3H, s, OMe), 2.45 (3H, s, Me). ^31^P NMR (CDCl_3_, 202 MHz) δ-143.11 (q, ^1^*J_P-F_* = 759.33 Hz). Anal. found; C, 48.2; H, 3.7; N, 5.8%, C_29_H_26_F_6_N_3_O_3_PPd requires: C, 48.6; H, 3.6; N, 5.8%. **6c**: Yellow solid, yield 92%. IR (KBr) υC=N 1568 cm^−1^. ^1^H NMR (Acetone-d_6_, 300 MHz) δ(ppm): 9.00–8.10 (9H, m, phen, *H*C=N) 7.73 (2H, d, *N* = 8.9 Hz, Hb), 7.38 (2H, d, *N* = 8.9 Hz, Ha), 6.43 (1H, s, H5), 4.06 (3H, s, OMe), 3.97 (3H, s, OMe), 3.84 (3H, s, OMe). ^31^P NMR (acetone-d_6_, 202 MHz) δ-155.76 (q, ^1^*J_P-F_* = 754.93 Hz). Anal. found; C, 42.6; H, 3.0; N, 5.2%, C_28_H_23_BrF_6_N_3_O_3_PPd requires: C, 43.1; H, 3.0; N, 5.4%.

**Preparation of 7a–b**. 1,1-Bis(diphenylphosphino)ethene (0.07 mmol, 2 eq.) was added to a suspension of **2bCl** (0.035 mmol, 1eq.). The solution was stirred for 5 h at room temperature, and the solvent was removed under reduced pressure. The resulting yellow solid was dried under vacuum. **7a**: Brown solid, yield 60%. IR (KBr) υC=N 1580 cm^−1^. ^1^H NMR (CDCl_3_, 300 MHz) δ(ppm): 8.38 (1H, d, ^4^*J*(HiP) = 7.9 Hz, *H*C=N), 7.83–7.30 (20H, m, PPh_2_), 6.25 (2H, m, P(C=C*H*_2_)P), 6.01 (1H, dd, ^4^*J* (H5P) = 9.8, 7.4 Hz, H5), 3.97 (3H, s, OMe), 3.74 (3H, s, OMe), 3.20 (1H, m, N-C*H*Cy), 3.08 (3H, s, OMe), 2.38–0.34 (10H, m, Cy). ^31^P NMR (CDCl_3_, 202 MHz) δ 11,57 (d, ^2^*J_P-P_* = 15.6 Hz), -5.94 (d, ^2^*J_P-P_* = 15.7 Hz). Anal. found: C, 62.9; H, 5.2; N: 1.6%, C_42_H_44_ClNO_3_P_2_Pd requires: C, 61.9; H, 5.4; N, 1.7%. **7b**: Yield 77%. IR (KBr) υC=N 1581 cm^−1^. ^1^H NMR (CDCl_3_, 300 MHz) δ(ppm) = 8.38 (1H, d, ^4^*J*(HP) = 7.2 Hz, *H*C=N), 8.02–7.16 (20H, m; PPh_2_), 6.95 (2H, d, *N* = 8.1 Hz C_6_H_4_), 6.77 (2H, d, *N* = 8.1 Hz C_6_H_4_), 6.47–6.24 (2H, m, P(C=C*H*_2_)P), 6.05 (1H, dd, ^4^*J*(H5P)= 9.9, 7.3 Hz H5), 4.00 (3H, s, OMe), 3.77 (3H, s, OMe), 3.15 (3H, s, OMe), 2.20 (3H, s, Me). ^31^P NMR (CDCl_3_, 202 MHz) δ 7.49 (d, ^2^*J_P-P_* = 12.8 Hz), −9.86 (d, ^2^*J_P-P_* = 12.8 Hz). Anal. found: C, 62.1; H, 5.1; N: 1.5%, C_43_H_40_ClNO_3_P_2_Pd requires: C, 62.8; H, 4.9; N, 1.7%.

**Preparation of 8a–b**. vdpp (0.046 g, 0.117 mmol, 2 eq.) and NH_4_PF_6_ (0.037 g, 0.023 mmol, 4 eq.) were added a suspension of **2a–bCl** (0.060 mmol, 1 eq.) in acetone. The initial solution led to a yellow precipitate that was filtered off and dried under vacuum. **8a**: Yellow solid, yield 61%. IR (KBr) υC=N 1589 cm^−1^. ^1^H NMR (CDCl_3_, 300 MHz) δ 8.27 (2H, m, *H*C=N), 8.16–7.13 (20H, m, PPh_2_), 6.16 (2H, m, H5), 5.25–5.12 (2H, m, P(C=C*H*_2_)P), 4.32 (2H, s, N-C*H*Cy), 3.88 (3H, s, OMe), 3.64 (6H, s, OMe), 2.59 (6H, s, OMe), 2.47–0.99 (20H, m, Cy). ^31^P NMR (CDCl_3_, 202 MHz) δ 44.62 (bs). Anal. found: C, 55.1; H, 5.1; N, 2.0%, C_58_H_66_Cl_2_N_2_O_6_P_2_Pd_2_ requires, C, 56.5; H, 5.4; N, 2.3%. **8b**: Yield 43%. IR (KBr) υC=N 1575 cm^−1^. ^1^H NMR (CDCl_3_, 300 MHz) δ 8.34(2H, m, *H*C=N), 8.20–7.19 (28H, m, PPh_2_, C_6_H_4_), 6.20–5.98 (2H, m, P(C=C*H*_2_)P), 5.29, (2H, m, H5), 3.93 (6H, s, OMe), 3.66 (6H, s, OMe), 2.67 (6H, s, OMe), 2.38 (6H, s, Me). ^31^P NMR (CDCl_3_, 202 MHz) δ 43.37 (bs). Anal. found: C, 57.2; H, 5.2; N, 2.2%, C_60_H_58_Cl_2_N_2_O_6_P_2_Pd_2_ requires, C, 57.7; H, 4.7; N, 2.2%.

**Preparation of 9b**. 1,1′-Bis(diphenylphosphino)ferrocene (0.029 g, 0.07 mmol, 2 eq.) was added to a suspension of 2bCl (0.03 g, 0.035 mmol). The solution was stirred for 4 h at room temperature, and the solvent was removed under reduced pressure. The solvent was removed, and the residue was recrystallized with dichloromethane/hexane to provide an orange solid. Yield 76%. IR (KBr) υC=N 1578 cm^−1^. ^1^H NMR (CDCl_3_, 300 MHz) δ 8.28 (1H, d, ^4^*J*(HP) = 9.3 Hz, *H*C=N), 7.68–7.18 (24H, m, PPh_2_, C_6_H_4_), 5.72 (1H, d, ^4^*J*(H5P) = 6.3 Hz, H5), 5.22–4.21 (8H, m, Cp), 3.91 (3H, s, OMe), 3.83 (3H, s, OMe), 2.94 (3H, s, OMe), 2.59 (3H, s, Me). ^31^P NMR (CDCl_3_, 202 MHz) δ 29.40 (d, *J* = 31.0 Hz), 27.64 (d, *J* = 30.9 Hz), −145.82 (q, 710.3 Hz). Anal. found: C, 62.9; H, 4.9; N, 1.1%, C_51_H_46_ClFeNO_3_P_2_Pd requires: C, 62.5; H, 4.7; N, 1.4%.

### 4.2. Crystal Structure Analysis and Details on Data Collection and Refinement

See Supporting Information.

## Figures and Tables

**Table 1:** Reaction conditions ^a^ for the SMC.

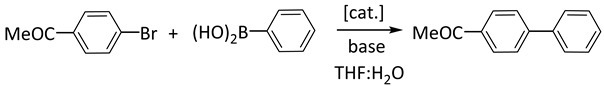									
Entry	[cat.]	T (h)	T (°C)	Yield ^c^	Entry	[cat.]	T (h)	T (°C)	Yield ^c^
**1**	**1b**	5	80	100	**20**	**4b**	5	rt	47
**2**	**1c**	5	80	98	**21**	**4c**	5	rt	52
**3**	**2aCl**	5	80	100	**22**	**5a**	24	40	18
**4**	**2aBr**	0.5	80	100	**23**	**5a**	24	80	100
**5**	**2aBr**	2	rt	100	**24**	**5a**	24	rt	0
**6**	**2bCl**	5	80	100	**25**	**5b**	24	80	66
**7**	**2bCl**	1	rt	80	**26**	**5b**	24	rt	0
**8**	**2bBr**	1	rt	83	**27**	**6a**	24	80	33
**9**	**2cCl**	2	rt	88	**28**	**6a**	24	rt	0
**10**	**3a**	1	80	100	**29**	**6b**	24	80	20
**11**	**3a**	1	rt	35	**30**	**6b**	24	rt	0
**12**	**3a**	3	rt	100	**31**	**7a**	5	80	100
**13**	**3b**	1	rt	37	**32**	**7a**	24	rt	99
**14**	**3b**	3	rt	90	**33**	**7b**	5	80	100
**15**	**3b**	1	rt	37	**34**	**7b**	24	rt	95
**16**	**3b**	3	rt	94	**35**	**8a**	5	80	95
**17**	**4a**	2	80	83	**36**	**8b**	5	80	90
**18**	**4a**	24	rt	100	**37**	**8b**	24	rt	3
**19**	**4b**	5	80	90	**38**	**9b**	24	80	15

^a^ Reaction conditions: 0.1 mmol 4-bromoacetophenone, 1.2 eq. phenylboronic acid, 2 eq. base K_2_CO_3_, 2% mol catalyst; 3 cm^3^ THF:H_2_O (2:1), 2 mol% cat.; ^c^ determined by NMR.

**Table 2:** Catalytic activity of compound **2bCl** under different reaction conditions ^a^.

Entry	Base	Solvent ^b^	T (°C)	Yield ^c^
**1**	K_2_CO_3_	Toluene	80	65
**2**	K_2_CO_3_	THF:H_2_O ^d^	80	100
**3**	K_2_CO_3_	H_2_O	80	91
**4**	K_2_CO_3_	EtOH:H_2_O ^e^	80	100
**5**	K_2_CO_3_	THF:H_2_O ^c^	rt	41
**6**	K_3_PO_4_	THF:H_2_O ^c^	rt	41
**7**	K_2_CO_3_	EtOH:H_2_O ^d^	rt	100
**8**	K_3_PO_4_	EtOH:H_2_O ^d^	rt	100

^a^ Reaction conditions: 1 mmol 4-bromoacetophenone, 1.2 eq. phenylboronic acid, 2 eq. base K_2_CO_3_, 2% mol **2bCl**; ^b^ 2 cm^3^; ^c^ determined by NMR; ^d^ THF:H_2_O (2:1); ^e^ EtOH:H_2_O (1:1).

**Table 3:** Catalytic activity of compound **2bCl** ^a^.

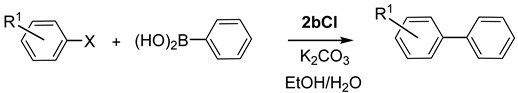						
Entry	Aryl Halide	Product	No.	T (h)	T (°C)	Yield ^b^
**1**	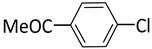	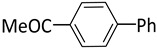	**1**	24	100	98
**2**	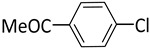	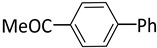	**1**	24	rt	57
**3**	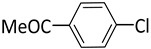	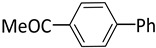	**1**	48	rt	64
**4**	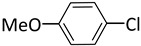	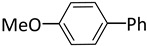	**2**	24	100	13
**5**	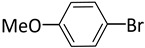	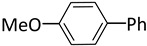	**2**	24	rt	100
**6**	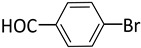	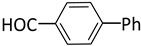	**3**	1	100	100
**7**	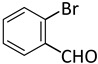	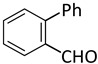	**4**	1	100	100
**8**	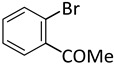	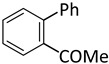	**5**	1	100	100
**9**	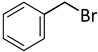	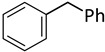	**6**	4	100	98
**10 ^c^**	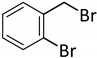	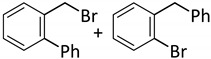	**7**	4	100	60/33 (7/8)
**11 ^c^**	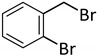	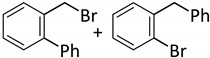	**7**	24	100	60/33 (7/8)
**12 ^d^**	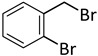	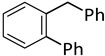	**8**	4	100	33/62 (8/7)
**13 ^d^**	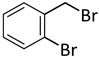	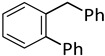	**8**	24	100	53/38 (8/7)
**14 ^d^**	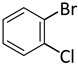	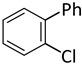	**9**	2	100	88

^a^ Reaction conditions: 1 mmol aryl halide, 1.2 eq. phenylboronic acid, 2 eq. base K_2_CO_3_, 2% mol 2bCl; 2 cm^3^ EtOH:H_2_O (2:1), 2 mol% cat.; ^b^ determined by NMR; ^c^ 1 mmol phenylboronic acid; ^d^ 2.4 mmol phenylboronic acid.

**Table 4:** Catalyst comparison ^a^ for the SMC.

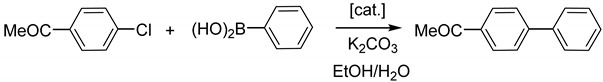					
Entry	[cat.]	[cat.] mol%	T (h)	T (°C)	Yield ^c^
**1**	**2bCl**	2%	24	100	98
**2**	**2bCl**	2%	48	rt	64
**3**	Pd(OAc)_2_	4%	24	100	64
**4** ^b^	Pd(OAc)_2_ + **b**	4%	24	100	80

^a^ Reaction conditions: 1 mmol 4-chloroacetophenone, 1.2 eq. phenylboronic acid, 2 cm^3^ EtOH:H_2_O (1:1); ^b^ Pd(OAc)_2_ 4% molar + ligand **b** 4% molar. ^c^ determined by NMR.

## Data Availability

Not applicable.

## Supplementary Materials

The following supporting information can be downloaded at: https://www.mdpi.com/article/10.3390/molecules27103146/s1. CCDC 2162245 for compound 2cCl. CCDC 2162246 for compound 1c.
