# Supplementary material for: Imine Palladacycles: Synthesis, Structural Analysis and Application in Suzuki–Miyaura Cross Coupling in Semi-Aqueous Media
Source: Molecules. 2022 May 14;27(10):3146. doi: 10.3390/molecules27103146 (PMC9144456; doi:10.3390/molecules27103146)
Supplement: Supplementary file 1 [file molecules-27-03146-s001.zip › JM VILA SI.pdf]

1c

Table S1. Crystal data and structure refinement for **1c.**

Identification code	**1c**
Empirical formula	$C_{36}H_{34}Br_2N_2O_{10}Pd_2$ [+ solvent]
Formula weight	1025.341
Temperature/K	99.99
Crystal system	triclinic
Space group	P-1
a/Å	11.688(5)
b/Å	13.069(5)
c/Å	13.658(5)
α/°	97.379(5)
β/°	97.048(5)
γ/°	107.028(5)
Volume/Å^3	1949.8(13)
Z	2
ρ_{calc}g/cm^3	1.746
μ/mm^{-1}	3.026
F(000)	1007.8
Crystal size/mm^3	0.4 × 0.23 × 0.16
Radiation	Mo Kα (λ = 0.71073)
2Θ range for data collection/°	3.06 to 52.8
Index ranges	-14 ≤ h ≤ 14, -14 ≤ k ≤ 16, -17 ≤ l ≤ 16
Reflections collected	28912
Independent reflections	7955 [R_{int} = 0.0381, R_{sigma} = 0.0481]
Data/restraints/parameters	7955/0/477
Goodness-of-fit on F^2	1.020
Final R indexes [I>=2σ (I)]	R_1 = 0.0314, wR_2 = 0.0579
Final R indexes [all data]	R_1 = 0.0458, wR_2 = 0.0622
Largest diff. peak/hole / e Å^{-3}	1.09/-0.78

Table S2. Bond Lengths for 1c.

Atom	Atom	Length/Å	Atom	Atom	Length/Å
Pd1	Pd2	2.9114(10)	N2	C24	1.430(4)
Pd1	O7	2.140(2)	C1	C2	1.419(4)
Pd1	O9	2.045(2)	C1	C6	1.403(4)
Pd1	N1	2.027(3)	C1	C7	1.421(4)
Pd1	C6	1.962(3)	C2	C3	1.382(4)
Pd2	O8	2.041(2)	C4	C3	1.410(4)
Pd2	O10	2.141(2)	C4	C5	1.391(4)
Pd2	N2	2.045(3)	C6	C5	1.378(4)

Atom	Atom	Length/Å	Atom	Atom	Length/Å
Pd2	C22	1.962(3)	C8	C13	1.379(4)
Br1	C11	1.905(3)	C9	C8	1.395(4)
Br2	C27	1.903(3)	C10	C9	1.383(4)
O1	C2	1.366(4)	C11	C10	1.381(4)
O1	C14	1.441(4)	C11	C12	1.379(4)
O2	C3	1.383(4)	C12	C13	1.395(4)
O2	C15	1.432(4)	C17	C18	1.406(4)
O3	C4	1.354(4)	C17	C22	1.401(4)
O3	C16	1.423(4)	C19	C18	1.383(4)
O4	C18	1.380(4)	C19	C20	1.407(5)
O4	C30	1.436(4)	C21	C20	1.383(5)
O5	C19	1.379(4)	C22	C21	1.387(4)
O5	C31	1.431(4)	C23	C17	1.423(4)
O6	C20	1.369(4)	C25	C24	1.384(4)
O6	C32	1.429(4)	C25	C26	1.387(4)
O7	C33	1.248(4)	C27	C26	1.391(5)
O8	C33	1.272(4)	C28	C27	1.376(5)
O9	C35	1.275(4)	C28	C29	1.390(4)
O10	C35	1.243(4)	C29	C24	1.391(4)
N1	C7	1.303(4)	C33	C34	1.508(4)
N1	C8	1.429(4)	C35	C36	1.505(5)
N2	C23	1.298(4)			

Table S3. Bond Angles for **1c.**

Atom	Atom	Atom	Angle/°	Atom	Atom	Atom	Angle/°
O7	Pd1	Pd2	76.35(5)	C6	C5	C4	119.8(3)
O7	Pd1	O9	89.04(8)	C1	C6	Pd1	113.1(2)
O9	Pd1	Pd2	86.95(6)	C5	C6	Pd1	127.3(2)
N1	Pd1	Pd2	95.12(7)	C5	C6	C1	119.6(3)
N1	Pd1	O7	98.09(9)	C1	C7	N1	116.8(3)
N1	Pd1	O9	172.86(9)	C9	C8	N1	118.1(3)
C6	Pd1	Pd2	105.64(8)	C13	C8	N1	121.8(3)
C6	Pd1	O7	177.91(10)	C13	C8	C9	120.1(3)
C6	Pd1	O9	91.70(11)	C8	C9	C10	120.0(3)
C6	Pd1	N1	81.16(12)	C9	C10	C11	118.8(3)
O8	Pd2	Pd1	85.57(6)	C10	C11	Br1	118.9(2)
O10	Pd2	Pd1	74.95(6)	C12	C11	Br1	119.0(2)
O10	Pd2	O8	89.43(9)	C12	C11	C10	122.1(3)
O10	Pd2	N2	98.08(9)	C13	C12	C11	118.5(3)
N2	Pd2	Pd1	98.38(7)	C8	C13	C12	120.2(3)
N2	Pd2	O8	172.19(10)	C18	C17	C23	124.3(3)
C22	Pd2	Pd1	109.62(9)	C22	C17	C18	120.6(3)

Atom	Atom	Atom	Angle/°	Atom	Atom	Atom	Angle/°
C22	Pd2	O8	90.78(11)	C22	C17	C23	115.1(3)
C22	Pd2	O10	175.43(10)	C17	C18	O4	117.8(3)
C22	Pd2	N2	81.56(12)	C17	C18	C19	119.6(3)
C2	O1	C14	119.6(2)	C19	C18	O4	122.4(3)
C15	O2	C3	112.6(2)	C18	C19	O5	120.7(3)
C16	O3	C4	117.3(2)	C20	C19	O5	120.2(3)
C18	O4	C30	116.1(2)	C20	C19	C18	119.1(3)
C31	O5	C19	113.0(2)	C19	C20	O6	114.1(3)
C20	O6	C32	117.4(3)	C21	C20	O6	124.5(3)
C33	O7	Pd1	129.59(19)	C21	C20	C19	121.4(3)
C33	O8	Pd2	122.1(2)	C20	C21	C22	119.7(3)
C35	O9	Pd1	120.2(2)	C17	C22	Pd2	112.9(2)
C35	O10	Pd2	131.6(2)	C21	C22	Pd2	127.5(2)
C7	N1	Pd1	113.7(2)	C21	C22	C17	119.6(3)
C8	N1	Pd1	126.5(2)	C17	C23	N2	117.5(3)
C8	N1	C7	119.7(3)	C25	C24	N2	119.9(3)
C23	N2	Pd2	112.9(2)	C29	C24	N2	119.7(3)
C24	N2	Pd2	128.0(2)	C29	C24	C25	120.3(3)
C24	N2	C23	118.9(3)	C24	C25	C26	119.9(3)
C6	C1	C2	121.1(3)	C25	C26	C27	119.2(3)
C7	C1	C2	124.3(3)	C26	C27	Br2	119.7(3)
C7	C1	C6	114.6(3)	C26	C27	C28	121.4(3)
C1	C2	O1	115.9(3)	C28	C27	Br2	119.0(3)
C3	C2	O1	125.4(3)	C29	C28	C27	119.2(3)
C3	C2	C1	118.6(3)	C24	C29	C28	120.0(3)
C2	C3	O2	121.6(3)	O7	C33	O8	126.4(3)
C2	C3	C4	119.7(3)	C34	C33	O7	118.6(3)
C4	C3	O2	118.7(3)	C34	C33	O8	115.0(3)
C3	C4	O3	114.0(3)	O10	C35	O9	126.3(3)
C3	C4	C5	121.2(3)	C36	C35	O9	115.8(3)
C5	C4	O3	124.9(3)	C36	C35	O10	117.9(3)

2cCl

Table S4. Crystal data and structure refinement for 2cCl

Identification code	**2cCl**
Empirical formula	$C_{64}H_{60}Br_4Cl_4N_4O_{12}Pd_4$
Formula weight	1964.295
Temperature/K	99.99
Crystal system	monoclinic
Space group	$P2_1/c$
a/Å	13.9156(3)

b/Å	3.92009(8)
c/Å	30.0833(6)
α/°	90
β/°	102.0378(10)
γ/°	90
Volume/Å^3	1604.97(6)
Z	1
ρ_{calc}g/cm^3	2.032
μ/mm^{-1}	3.825
F(000)	956.3
Crystal size/mm^3	0.1 × 0.06 × 0.04
Radiation	Mo Kα (λ = 0.71073)
2Θ range for data collection/°	3 to 53.22
Index ranges	-17 ≤ h ≤ 17, -4 ≤ k ≤ 4, -37 ≤ l ≤ 37
Reflections collected	21117
Independent reflections	3313 [R_{int} = 0.0501, R_{sigma} = 0.0387]
Data/restraints/parameters	3313/0/211
Goodness-of-fit on F^2	1.036
Final R indexes [I>=2σ (I)]	R_1 = 0.0283, wR_2 = 0.0501
Final R indexes [all data]	R_1 = 0.0448, wR_2 = 0.0541
Largest diff. peak/hole / e Å^{-3}	0.81/-0.88

Table S5. Bond Lengths for 2cCl.

Atom	Atom	Length/Å	Atom	Atom	Length/Å
Pd1	Cl1[1]	2.4485(8)	C2	C1	1.404(4)
Pd1	Cl1	2.3338(8)	C2	C3	1.389(4)
Pd1	N1	2.048(3)	C3	C4	1.402(5)
Pd1	C6	1.966(3)	C4	C5	1.398(4)
Br2	C11	1.902(3)	C6	C1	1.404(4)
O1	C2	1.376(4)	C6	C5	1.375(4)
O1	C14	1.433(4)	C7	C1	1.422(4)
O2	C3	1.383(4)	C8	C13	1.392(4)
O2	C15	1.441(4)	C9	C8	1.388(4)
O3	C4	1.365(4)	C9	C10	1.383(4)
O3	C16	1.427(4)	C11	C10	1.378(4)
N1	C7	1.304(4)	C12	C11	1.389(5)
N1	C8	1.423(4)	C12	C13	1.388(4)

Table S6. Bond Angles for 2cCl.

Atom	Atom	Atom	Angle/°	Atom	Atom	Atom	Angle/°
N1	Pd1	Cl1	173.79(7)	C4	C3	C2	119.0(3)
N1	Pd1	Cl1[1]	99.35(7)	C3	C4	O3	115.5(3)

Atom	Atom	Atom	Angle/°	Atom	Atom	Atom	Angle/°
C6	Pd1	Cl1	93.83(9)	C5	C4	O3	123.3(3)
C6	Pd1	Cl1[1]	178.38(9)	C5	C4	C3	121.2(3)
C6	Pd1	N1	81.20(11)	C4	C5	C6	119.9(3)
C14	O1	C2	115.1(3)	C1	C6	Pd1	113.0(2)
C15	O2	C3	114.5(2)	C5	C6	Pd1	127.6(2)
C16	O3	C4	116.8(2)	C5	C6	C1	119.4(3)
C7	N1	Pd1	113.2(2)	C1	C7	N1	117.0(3)
C7	N1	C8	118.9(3)	C9	C8	N1	121.4(3)
C8	N1	Pd1	127.9(2)	C13	C8	N1	118.7(3)
C6	C1	C2	120.9(3)	C13	C8	C9	119.8(3)
C7	C1	C2	123.8(3)	C10	C9	C8	120.3(3)
C7	C1	C6	115.1(3)	C11	C10	C9	119.4(3)
C1	C2	O1	118.3(3)	C10	C11	Br2	118.2(3)
C1	C2	C3	119.6(3)	C10	C11	C12	121.3(3)
C3	C2	O1	122.0(3)	C12	C11	Br2	120.4(2)
C2	C3	O2	119.5(3)	C13	C12	C11	119.0(3)
C4	C3	O2	121.3(3)	C12	C13	C8	120.1(3)
