# Supplementary material for: Imine Palladacycles: Synthesis, Structural Analysis and Application in Suzuki–Miyaura Cross Coupling in Semi-Aqueous Media
Source: Molecules. 2022 May 14;27(10):3146. doi: 10.3390/molecules27103146 (PMC9144456; doi:10.3390/molecules27103146)

# Imine palladacycles: synthesis, structural analysis and applications to the Suzuki-Miyaura cross coupling in semi-aqueous media

Brais Bermúdez-Puente, Luis A. Adrio, Fátima Lucio-Martínez, Francisco Reigosa, Juan M. Ortigueira and José M. Vila*

Department of Inorganic Chemistry, University of Santiago de Compostela, E-15782, Santiago de Compostela (Spain).

Imine palladacycles are useful as catalysts in the Suzuki-Miyaura cross coupling reaction in aqueous and semi-aqueous media.

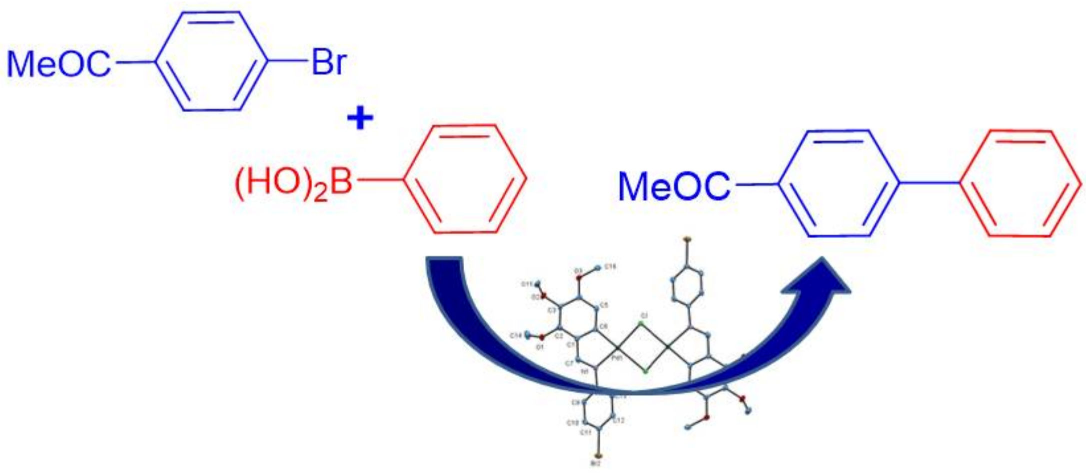

Supplement: Supplementary file 1 [file molecules-27-03146-s001.zip › JM VILA TABLE OF CONTENTS.pdf]
